# Camel-Related Facial Injuries: A Seven-Year Retrospective Study

**DOI:** 10.3390/clinpract13040081

**Published:** 2023-08-01

**Authors:** Mohamed A. Al-Ali, Hussam M. Mousa, Isabelle Nibelle, Ashraf F. Hefny

**Affiliations:** 1 Department of Surgery, College of Medicine and Health Sciences, United Arab Emirates University, Abu Dhabi, United Arab Emirates; m.al-ali@uaeu.ac.ae (M.A.A.-A.); hmousa@uaeu.ac.ae (H.M.M.); 2 Department of Otolaryngology, Al Ain Hospital, Abu Dhabi, United Arab Emirates; isabellenibelle@gmail.com

**Keywords:** camel, epidemiology, face, injury

## Abstract

Facial injuries caused by camels can be associated with adverse long-term effects on patients’ quality of life. We aimed to investigate camel-related facial injuries in Al-Ain City, UAE, focusing on their incidence, types, mechanisms, anatomical distribution, and outcomes, to enhance preventive measures. We retrospectively collected data from all patients who were admitted to our hospital with camel-related facial injuries from January 2014 through January 2021. Thirty-six patients were included; all were males, with a mean (range) age of 31 (14–66) years, 29 (80.5%) were camel caregivers. The most common mechanisms of injury were falling while riding a camel and camel kicks. The head was the most commonly injured region in 52.7%. Twenty-three (63.8%) patients had facial bone fractures. The middle third of the face accounted for 71.4% of the bony fractures. The most performed surgical procedures in our patients were soft tissue laceration repair and open reduction with internal fixation of fractures (ORIF). Camel-related facial injuries affect young adult male camel caregivers working on camel farms. Orbital and maxillary bone fractures are the most predominant fractures requiring operative management. Legislation for compulsory helmet usage may reduce the incidence of these injuries and their serious consequences.

## 1. Introduction

Interactions between humans and animals can result in different types of injuries to humans. These injuries vary depending on the animal’s type, size, and behavior [1]. Worldwide, injuries caused by large animals, including camels, are associated with substantial morbidity and mortality [2,3].

Camels are domestic animals in many areas of the world, including the UAE. More than 300,000 camels are already registered in Dubai city [4]. For the local population, these camels hold significant value as source of food, milk, transport, and wealth. In addition, camel racing is one of the most famous traditional sports in the UAE. Eid et al. showed that camel-related injuries accounted for 84.3% of animal-related injuries in the UAE [1]. Owing to the immense force involved, injuries sustained from camels are considered high-energy trauma. 

Previous reports have shown that the head and face were the second most common camel-related injuries following the upper and lower extremities [1]. Facial injury may have adverse long-term effects on a patient’s quality of life. These injuries may include soft tissue lacerations and facial bone fractures that often require surgical management [5,6]. Nevertheless, the injuries may have psychological impacts, related to cosmetic disfigurement, and increased demand for aesthetics [7].

The epidemiology of camel-related injuries varies depending on the social and cultural aspects of the studied population [8]. 

Despite the apparent potential to produce a variety of serious injuries, there remains a scarcity of research regarding camel-related facial injuries (CRFIs). We have previously described the biomechanics and severity of head, face, and neck camel-related injuries in different sets of patients at different times [5]. The current study has focused explicitly on CRFIs and their management at our institution. Therefore, the overlap with the formerly published paper is minor. We aimed to investigate camel-related facial injuries in Al-Ain City, UAE with a focus on their incidence, types, injury mechanisms, anatomical distribution, and outcomes. This information can be used to develop preventive measures, such as protective gear or safety protocols, specifically tailored to minimize the risk of facial injuries in vulnerable areas and will provide valuable insights into the effectiveness of current practices.

## 2. Methods 

### 2.1. Ethical Considerations

Ethical approval for this study was obtained from AAH Research Ethics Governance Committee, Al Ain Hospital, Al Ain, Abu Dhabi, UAE (AAHEC-04-18-086). Written informed consent was taken from the patients who agreed to the publication of their clinical data. Specific consent was also obtained from one patient for the publication of identifying figures/images. In addition, all methods were conducted according to the relevant guidelines and regulations. 

### 2.2. Data Collection

We conducted a retrospective analysis utilizing prospectively collected data from Al Ain Hospital Trauma Registry. All patients with CRFIs who were admitted for more than one day or those who passed away following their arrival at the hospital during the period from January 2014 to January 2021 were studied. Al-Ain Hospital is a university-affiliated community-based hospital with trauma and acute care facilities. It is located in Al Ain city, which has a population of 766,936 inhabitants [9]. 

The details of facial injuries were studied using a specially designed study protocol. In addition, we assessed the severity of the injury of the different anatomical regions using the Abbreviated Injury Severity Score (AIS). Overall injury severity was determined using the Injury Severity Score (ISS). Both were calculated manually using the AIS 2008 handbook [10]. 

Data collected included demography, vital signs, Glasgow Coma Score (GCS) on admission, anatomical location and severity of the injury, associated injuries, and surgical management. All patients underwent an assessment to determine the primary mechanism of injury, representing the initial force that directly caused the injury. Any secondary mechanisms from additional factors or influences were also determined if applicable.

The patients were followed up during their hospital stay to record the length of hospital stay (LOS), complications, and outcomes. Face injuries were classified into soft tissue and facial bone fractures. All facial fractures were classified and confirmed by preoperative computed tomography (CT) imaging and operative findings. Patients with facial fractures were classified depending on the affected region of the face into the upper third, middle third, and lower third. 

Patients with facial injuries that resulted in significant damage, such as facial fractures, severe soft tissue trauma, injuries that affected critical facial structures, or significant associated injuries, were offered operative management.

### 2.3. Statistical Analysis

The collected data were entered into a Microsoft Excel spreadsheet (Microsoft Corporation, Seattle, WA, USA). A simple descriptive statistical analysis was performed. Data were presented as median (range) or number (%) as appropriate. Statistical analyses were performed using the Statistical Package for the Social Sciences (IBM-SPSS version 26, Chicago, IL, USA).

## 3. Results

During the study period, a total of 98 patients with camel-related injuries required admission to the hospital. The estimated incidence of hospitalized patients with camel-related injuries in general in Al-Ain city was 2.1 per 100,000 population per year. 

Thirty-six (36.7%) of the admitted patients with camel-related injuries sustained facial injuries and were included in the study. All included patients were males with a mean (range) age of 31 (14–66) years. Pakistani nationality was the most commonly injured nationality (15 patients, 41.7%), followed by Bangladeshi nationality (6 patients, 16.7%) and UAE nationality (4 patients, 11.1%). The estimated incidence of hospitalized patients with CRFI was 0.67 per 100,000 per year. 

None of our patients had used a helmet or any other protective gear at the time of injury. 

Twenty-nine (80.6%) patients were camel caregivers, six (16.7%) were car drivers involved in road traffic collisions with camels, and one patient (2.8%) was a camel jockey. 

Twenty-nine (80.6%) patients were injured on the farm, five (13.9%) on the highway, and two (5.6%) on the racing track. Falling while riding a camel and camel kick were the most common mechanisms of injury in eleven patients for each (30.6%) (Table 1).

There were no significant statistical differences in the primary mechanisms regarding AIS (*p* = 0.262 Fisher’s Exact Test), GCS (*p* = 0.536 Fisher’s Exact Test), and ISS (*p* = 0.531 Fisher’s Exact Test). In five (13.9%) patients, a secondary mechanism of injury was observed. 

The median (range) ISS was 4.5 (1–29), and the median (range) GCS was 15 (10–15).

Twenty-three (63.9%) patients had an injury to another body region besides the face. The head was the most injury-associated region in 19 (52.8%) patients, followed by the upper limb in five (13.9%) and the neck in four (11.1%) patients. 

Thirty-two (88.9%) patients sustained soft tissue injuries and 23 (63.9%) patients had facial fractures. A total of 42 facial fractures were identified. Camel kick was the most common mechanism of facial fracture in 13 (56.5%) of those patients with facial fractures. The distribution of camel-related facial fractures by anatomical region is shown in Table 2.

**Figure 1:**
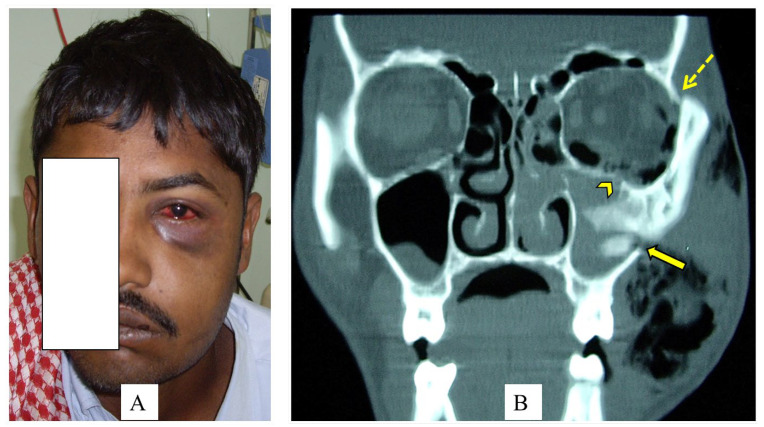
A 28-year-old camel caregiver was kicked in his face by a camel’s front leg (**A**). The left side of the face is swollen with clinically apparent subcutaneous emphysema, periorbital edema, ecchymosis, and subconjunctival hemorrhage. The coronal view CT scan of the facial bone (**B**) shows a fracture of the left superolateral orbital rim (hashed arrow), a comminuted fracture through the left orbital floor (arrowhead), and a fracture of the lateral wall of the left maxillary sinus (arrow). Specific consent has been obtained from the patient for the publication of identifying figures/images.

The most injured region was the middle third of the face, which accounted for 16 (69.6%) of the bony fractures. Maxillary bone and orbital bone fractures were the most frequent midfacial fractures (Figure 1). In nine patients with orbital fractures, orbital floor fractures, in five (55.6%) patients, were the most common type of fracture.

Nineteen (52.7%) patients required operative management for their injuries. The median time to surgery following hospital admission was 15 h. The most commonly performed surgical procedures were soft tissue laceration repair in eight (22.2%) patients, followed by open reduction with internal fixation of fractures (ORIF) in seven (19.4%) patients, and closed reduction of facial fractures in four (11%) patients. 

Patients stayed for a median (range) of 3 (1–13) days in the hospital. Two (5.6%) patients were intubated in the Emergency Room. One patient was admitted to the Intensive Care Unit (ICU), he had an associated severe head injury with skull fracture and epidural hematoma, and subsequently underwent craniotomy. There was no mortality among the patients. 

The patients were followed up for a median (range) of 7 (1–180) days. Five (13.9%) patients developed complications, including inferior alveolar nerve paresthesia in two patients (out of the six patients who had mandibular fractures), malunion in one patient, and epidural hematoma in another. After being repeatedly bitten by a camel, one patient had multiple complications to his face, including unilateral blindness, facial nerve paralysis, and a parotid fistula.

## 4. Discussion

The study has shown that about a third of the admitted cases of camel-related injuries involved the face. Similarly, a study by Caglayan et al. [11] showed that 43.9% of all large animal-related injuries affected the maxillofacial area, and horses were the most common animal involved (71.3%).

As demonstrated in past studies on camel-related injuries, all patients in the current study were males, most being young camel caregivers (80.6%) from the Indian subcontinent [1,5]. This is in contrast to reports on horse-related injuries that showed that injuries were more prominent among females [11,12,13]. Camel caregiver populations are low-income workers with low educational levels but with a good background and some skills in animal care in their home countries. They live on private camel farms in the hot desert away from the city. Surprisingly, none of the patients used any face and/or head protective gear at the time of injury. This might be due to a lack of awareness among those young camel caregivers about the value of wearing protective equipment during their work on the farms. Camel-related injuries are considered work-related injuries. Barss et al. [14] showed that animals caused 7% of occupational injuries in the UAE; 85% of these involved camels.

A previous study by Cunningham and Agel [15], which extensively assessed 512 patients with horse-related injuries, showed that 74.8% of patients who sustained injuries to the head were not wearing a helmet at the time of injury. Moreover, another study observed a lower prevalence and severity of facial fractures in patients who had worn helmets than in patients who did not [16]. 

The most common mechanisms of injury were falling from a camel on the face and camel kick, which were equally reported in our series (30.6%). Previous studies showed that animal kicks caused most animal-related injuries [5,12]. In contrast, Moss et al. showed that a fall was the leading mechanism of injury among horse riders [17]. In the current study, 19.4% of the patients were injured due to camel car collisions, especially on the highways. Stray camels tend to unexpectedly cross roads, which can be difficult to visualize at night by speeding drivers, leading to severe and fatal injuries. Warning signals on roads and fencing highways in areas known to have camels could help in reducing collisions with camels [13,18]. 

The study has shown no statistically significant difference between AIS, GCS, and ISS regarding different primary mechanisms of trauma. The presence of a secondary mechanism of injury could increase injury complexity and severity [18]. 

CRFI can range from minor soft tissue injuries to more complex injuries like multiple facial fractures. A previous study showed that 5.5% of facial fractures in the UAE are caused by camel kicks and bites [19]. In the current study, 23 (63.9%) patients sustained facial fractures, with a camel kick being the most common causative mechanism. This finding agrees with several studies that indicated that face injuries caused by camel and horse kicks resulted in fractures more frequently than with other mechanisms [13,18]. Camels may kick at high speed with their front knees or back hoof, transmitting the large force upon impact to skeletal structures. In keeping with other studies, the midface was the most commonly involved region [18]. The maxilla and the orbital floor were more susceptible to fracture than other sites in our series. In comparison, mandibular fractures were observed to be the most common facial bone fracture in other studies of large animal-related injuries [20,21]. 

Associated injuries to other body regions are commonly found among patients who have sustained facial injuries. In the present study, 63.9% of the patients had associated injuries, mostly affecting the head, which is consistent with previous studies [17,20,22].

In contrast, other studies showed that the upper extremity was the body region most commonly associated with injuries caused by large animals [23]. Ueeck et al. [13] showed that 74% of the patients with maxillofacial horse-related injuries had other associated injuries, commonly head or upper extremity. Researchers have also shown that facial injuries increase the risk of sustaining head and brain injuries [24,25]. Another study showed that patients with facial fractures to the midface had a significant association with traumatic brain injuries [26]. In our series, one patient developed epidural hematoma after sustaining multiple facial fractures to the upper and middle third of the face. Associated injuries correlated significantly with trauma mechanisms [27]. Similar to others, this study showed that associated head injury occurred commonly in patients who fell from or were kicked by a camel [5]. In addition, studies showed that associated head injury was the main cause of mortality among patients with large animal-related injuries [22,23,24,25,26,27,28]. Hence, in patients with facial injuries, diagnosing concomitant occult, potentially life-threatening injuries to other body regions is crucial.

Management of patients with facial injuries depends on different factors, including injury severity, surgeons’ preference, and resource availability. More than half of the patients in this study required operative management of their injury. In agreement with others, the most common procedures performed on our patient were repairing the facial lacerations and ORIF of facial fractures [24,27].

In the present study, our figure for the overall median length of hospital stay was lower than that reported by other studies on camel-related injuries [5,18]. Studies have shown that the most reliable predicting factors for mortality among patients with facial injuries were advanced age, increased ISS, and low GCS [25,29,30]. Our patients had a good overall outcome with no reported mortality. This good outcome may be related to the young age of our patients, with the median ISS being 4.5 and GCS being 15. Moreover, the majority had no severe concomitant injuries. In contrast, Hefny et al. [31] reported a mortality rate of 2.6%. Within their study, one patient died after a camel-related injury to the head, which was further complicated by intracranial injuries with subdural hematoma.

Late presentation and management following a traumatic injury are usually associated with increased clinical complications. However, most of our patients presented to the hospital shortly after the injury and were managed early, reflecting the low complication rate in our series.

Facial injuries caused by camels are mostly preventable. Effectively reducing the risk of these injuries will minimize the number of patients seeking treatment, reducing the burden of facial injuries for patients, their families, and the healthcare system. Most of the injuries in the present study were occupational injuries. Therefore, improving the safety culture and practices among camel caregivers, riders, and owners is essential. In addition, research and development involving better injury prevention materials and technology should be encouraged. Injury prevention strategies must educate camel caregivers about the proper handling of camels and modify the work environment with appropriate protective equipment. Protective gear can help in reducing the severity of the injury. Despite evidence from previous studies that using helmets reduced the severity of head injury among equestrians, general use remains low [12,31,32]. Furthermore, using helmets with an integrated face protector may have an additional benefit in preventing facial injuries. Unfortunately, to date, wearing protective safety gear at farms is not mandatory, and some private camel farms do not even provide any safety equipment. Although this study did not specifically assess the influence of helmet usage, it may be helpful to consider implementing legislation for compulsory helmet usage. Such measures can potentially reduce the incidence of these injuries and their serious consequences, as supported by other studies [12,32].

Inexperienced camel caregivers should be enlightened about the possible aggressive behavior of camels during the breeding season and the importance of using mouth muzzles to avoid camel bite injuries [6]. Stray camels should be prohibited from roaming on roads or residential areas to prevent camel–vehicle collisions. In addition, fencing highways in areas with camels could be an effective preventive measure [33]. 

We must acknowledge that our study has certain limitations. The sample size is small but is unique to the region. Owing to the retrospective nature of this study, our analysis may be subject to information bias and the potential for incomplete documentation. The rate of CRFIs in the present study may be underestimated. Our study did not include patients who died at the scene, those who were discharged from the Emergency Department without admission to the hospital, and those who did not reach our hospital because of treatment of minor injuries in other primary care clinics. Furthermore, the current study analyzed patients from a single trauma center, limiting its generalizability to the whole UAE. Nevertheless, the study has provided essential information that can be utilized by healthcare providers and guide authorities in establishing preventive measures to reduce camel-related injuries. 

Future research may focus on assessing the effectiveness of various preventive measures, such as protective gear, in reducing the incidence and severity of camel-related facial injuries. It would also be valuable to examine the long-term outcomes of individuals who have experienced these injuries, including the impact on quality of life and rehabilitation needs.

## 5. Conclusions

Camel-related facial injuries mainly affect camel caregivers. The main mechanisms of injury are falling from camels and camel kicks. Orbital and maxillary bone fractures are the most predominant fractures requiring operative management. Educating camel caregivers about camel behavior and handling and legislation for helmet usage are essential preventive steps. In addition, road safety measures such as warning signals and fencing could prevent collisions with camels.

## Figures and Tables

**Table 1:** Mechanism of injury in 36 patients with camel-related facial injuries during the period from January 2014 to January 2021, Al Ain Hospital, Al Ain, Abu Dhabi, UAE.

Mechanism of Injury	Primary Mechanism *n* (%)	Secondary Mechanism *n* (%)
Fall while riding	11 (30.6)	1 (2.8)
Camel kick	11 (30.6)	1 (2.8)
Camel–car collision	7 (19.4)	
Camel bite	4 (11.1)	
Blow by camel body	3 (8.3)	1 (2.8)
Stepped on by a camel		2 (5.6)

**Table 2:** Fracture subunit distribution in 23 patients with camel-related facial fracture injuries during the period from January 2014 to January 2021, Al Ain Hospital, Al Ain, Abu Dhabi, UAE.

Fracture	N (23)	% *
Orbital	9	39.1
Maxilla	9	39.1
Nasal bone	6	26.1
Zygoma	6	26.1
Mandible	6	26.1
Frontal bone	3	13
Dentoalveolar	2	8.7
Skull	1	4.4

* Patients may have more than one fractured bone; therefore, the total percentage adds up to more than 100%.

## Data Availability

The datasets generated during and/or analyzed during the current study are available from the corresponding author on request.

## Abbreviations

AIS	Abbreviated Injury Severity Score
CRFI	Camel-related Facial injury
GCS	Glasgow Coma Score
ICU	Intensive Care Unit
ISS	Injury Severity Score
LOS	Length of Stay
ORIF	Open Reduction with Internal Fixation
UAE	United Arab Emirates
